# Effects of legume-wheat rotation patterns on wheat yield, quality, and soil microbial community in the North China Plain

**DOI:** 10.3389/fmicb.2026.1764764

**Published:** 2026-02-12

**Authors:** Zhenwu Nan, Zhu Liu, Nana Xu, Kainan Zhao, Hongcui Dai, Yubin Wang, Weiwei Meng, Kaichang Liu

**Affiliations:** 1 National Engineering Research Center for Wheat and Maize, Crop Research Institute, Shandong Academy of Agricultural Sciences, Jinan, China; 2 Shandong Academy of Agricultural Sciences, Jinan, China

**Keywords:** legume rotation, quality, soil microbial community, wheat, yield

## Abstract

Incorporating legumes into crop rotation systems is an environmentally sound agricultural practice that improves soil quality and crop productivity. However, the mechanisms underlying these benefits, particularly the relationship between soil properties and microbial communities, remain unclear. This study evaluated the impact of three different rotation patterns, namely wheat-maize (WM), wheat-peanut (WP), and wheat-soybean (WS), on wheat yield, grain quality, soil physicochemical properties, soil enzyme activities, and microbial community. Our results demonstrated that legume-based rotations significantly improved wheat performance. In contrast to WM, WP consistently achieved the highest wheat yield, with a 3 year average increase of 10.7%. Furthermore, significant improvements in key quality parameters were observed in WP. Specifically, crude protein and wet gluten contents increased by 9.2% and 27.4%, respectively. These improvements were attributed to the enhancement of soil health. Legume rotations, particularly WP, led to significant improvements in soil total nitrogen (TN), available phosphorus (AP), and soil water content (SWC). This was accompanied by a strategic shift in soil enzymatic functions, with significantly higher activities of N-cycle (LAP, NAG) and P-cycle (ALP) enzymes. High-throughput sequencing revealed that legume rotations enriched specific bacterial phyla, such as Acidobacteria and Chloroflexi, and fostered a more complex and stable fungal co-occurrence network. Mantel test analysis revealed that wheat yield and quality were significantly correlated with several key soil parameters, including soil pH, TN, and the activities of NAG and ALP. PLS-PM analysis revealed that soil properties enhanced soil enzyme activity by shaping microbial communities, ultimately improving crop performance, demonstrating that microbial communities and soil enzyme activity play crucial roles. Collectively, these findings reveal that introducing legumes, especially peanut, boosts wheat yield and quality by enhancing soil nutrient availability and shaping a beneficial microbial community, serving as a sustainable strategy for wheat production.

## 1 Introduction

As China's producing region, the North China Plain (NCP) contributes approximately 45% of the national wheat output and 30% of the maize output (Zhao H. et al., 2022; Xia et al., 2023). The wheat-maize double-cropping rotation system is the most prevalent cropping practice in this region (Yang et al., 2023). Due to long-term wheat-maize (W-M) rotation and sustained reliance on heavy fertilizer and pesticide inputs to maintain high yields, soil quality is deteriorating (Yan et al., 2024). This not only threatens yield stability but also triggers a series of environmental pollution issues, such as soil acidification and nitrate leaching (Guo et al., 2010; Rosas et al., 2015). Incorporating legumes into crop rotation systems is recommended as a promising approach to maintain crop productivity and mitigate soil and environmental issues (Yu et al., 2014; Zhao J. et al., 2022).

The core advantage of legumes lies in their unique ability to fix nitrogen biologically. Through symbiosis with rhizobia, legumes convert atmospheric nitrogen into forms usable by plants (Qiao et al., 2024). Legume crops fix an estimated 33 Tg of nitrogen annually, accounting for 60% of all biological nitrogen fixation in agriculture (Reis Ely et al., 2025). Legumes not only require very little nitrogen themselves but also leave usable nitrogen for subsequent crops (Geng et al., 2023). Legumes can potentially provide subsequent grain crops with an estimated 60 kg N ha^−1^ (Preissel et al., 2015), thereby enabling these crops to achieve higher yields (Geng et al., 2023). A global-scale meta-analysis revealed that legume pre-crops not only increase the average yield of principal crops by 20.4%, but also demonstrate widespread effectiveness across diverse crops, soils, and climates (Zhao J. et al., 2022). In northern North America, planting wheat after leguminous crops results in a significant yield increase of up to 35% (Miller et al., 2002; Gan et al., 2015). Wheat-pea rotation significantly enhanced the yield stability of the system, which may be attributed to the strong biological nitrogen-fixing capacity, higher straw incorporation, and shallow root systems of pea (Liu et al., 2020). Therefore, incorporating legume crops into rotation systems could enhance system stability.

The composition of legume residues, rich in nitrogen, phosphorus, sugars, and proteins with a low C/N ratio, promotes rapid decomposition in soil (Yu et al., 2016). This process effectively drives nutrient cycling, thereby enhancing nutrient uptake for the subsequent crop (Kohmann et al., 2019). Mung bean-winter wheat rotation significantly increased wheat gluten protein content, substantially exceeding that of three cereal crop-based rotations (maize, sorghum, and millet) (Wang R. et al., 2025). A deeper understanding of how different legume-wheat rotation patterns affect wheat quality will provide scientific support for sustainably enhancing this cropping system.

Soil microbial communities are central to driving soil nutrient cycling and organic matter transformation, with their structure and function directly regulating soil fertility and ecosystem health (Bai et al., 2022). Changes in root exudates and soil nutrient release induced by crop rotation directly alter resource availability for soil microorganisms, reshaping the diversity and structure of bacterial and fungal communities (Ai et al., 2018). Compared to maize, wheat-soybean rotation increases soil pH and microbial biomass in the wheat rhizosphere, which benefits microbial decomposition, metabolic activity, and functional diversity (Song et al., 2022). By enhancing microbial biomass, promoting key enzyme activities, and alleviating microbial nitrogen limitation, the soybean-maize rotation drives nutrient cycling and organic carbon accumulation more efficiently, which ultimately improves overall soil functionality (Luo et al., 2024). However, how microbial community diversity and composition respond to different legume-wheat rotation combinations remains poorly understood.

This study describes experiments conducted in the NCP region from 2018 to 2021, involving the rotation of two legumes (soybean and peanut) with wheat. Based on the hypothesis that legume-wheat rotation influences crop performance through a cascade of soil and microbial changes, the objectives of this study were threefold: (1) to quantify the ultimate effects on wheat yield and quality; (2) to determine the effects of legume (soybean or peanut)-wheat rotations on key soil properties and enzyme activities; and (3) to elucidate how these rotations alter the soil microbial community and its co-occurrence network.

## 2 Materials and methods

### 2.1 Experimental site

**Figure 1:**
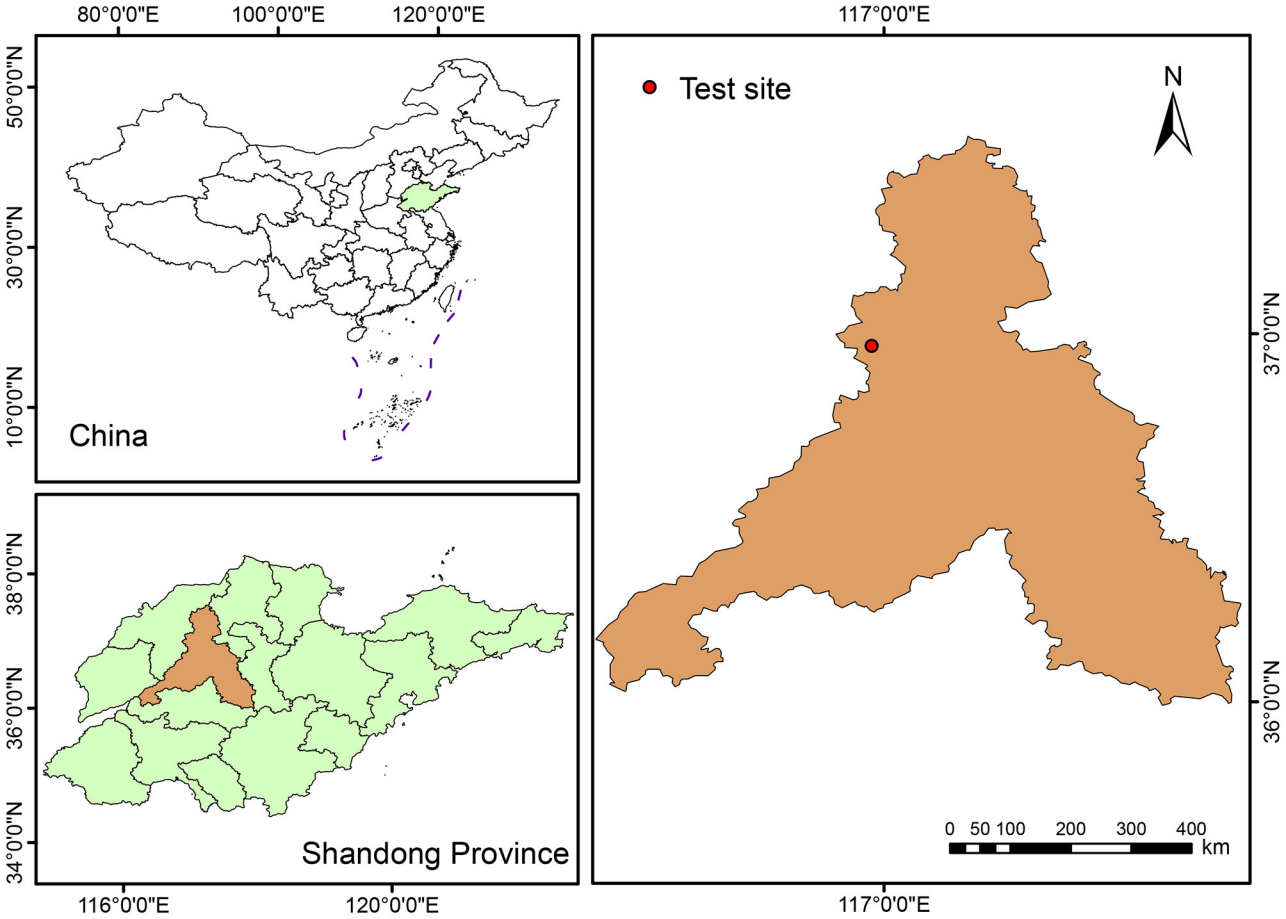
Location of experimental site in the North China plain.

The field experiment was conducted at the Comprehensive Experimental Base of Shandong Academy of Agricultural Sciences, located in Jiyang District (116°58′ E, 36°58′ N), Jinan, China (Figure 1). Situated on the alluvial plain of the lower Yellow River at an elevation of 20 m, the site features a warm-temperate, semi-humid monsoon climate. Key climatic characteristics include a mean annual temperature of 12.8 °C, annual precipitation of 580 mm, a 195-day frost-free period, and 2618 hours of annual sunshine. The soil is classified as a Luvisol derived from Yellow River alluvium, with a sandy loam texture in the topsoil. Prior to the experiment, baseline soil properties of the 0–20 cm layer were as follows: organic matter, 12.1 g kg^−1^; total nitrogen, 0.7 g kg^−1^; total phosphorus, 0.8 g kg^−1^; total potassium, 19.2 g kg^−1^; alkaline-hydrolyzable N, 50.6 mg kg^−1^; available P, 20.5 mg kg^−1^; available K, 151.9 mg kg^−1^; and a pH of 8.0. Prior to 2018, the area had consistently practiced a wheat-maize rotation system.

### 2.2 Experimental design

A long-term field experiment was initiated in 2018 using a randomized complete block design. The experiment consisted of three rotation systems, each replicated three times: wheat-maize (WM), wheat-peanut (WP), and wheat-soybean (WS). The crop varie-ties used were ‘Jimai 22′ for wheat (*Triticum aestivum* L.), ‘Denghai 605′ for maize (*Zea mays* L.), ‘Huayu 25′ for peanut (*Arachis hypogaea* L.), and ‘Qihuang 34′ for soybean (*Glycine max* L.). The fertilization regime was consistent across all treatments. A basal dose of compound fertilizer (N: P_2_O5: K_2_O = 15: 15: 15) was applied at a rate of 750 kg ha^−1^ before sowing each crop. Additionally, urea (46% N) was top-dressed at 225 kg ha^−1^ during the wheat jointing stage and the maize V12 stage. Wheat was typically sown in mid-October and harvested in early June of the following year. The summer crops (maize, peanut, and soybean) were sown in mid-June and harvested in early October. Other management practices follow conventional tillage methods, with straw incorporated into the soil.

### 2.3 Sample collection

During each harvest season from 2019 to 2021, a representative 15 m^2^ area was harvested from each of the three replicate plots at maturity to determine wheat yield. In March (jointing stage of wheat) and June (maturity stage of wheat) of 2021, soil samples were collected from the 0–20 cm soil layer using the five-point sampling method. For each sampling time, samples from the same plot were pooled into a single composite sample, with three replicates. All samples were collected in sterile self-sealing bags and immediately shipped on dry ice to the laboratory. Each sample was partitioned into two subsamples for different analyses. The first subsample, intended for DNA sequencing, was immediately stored at – 80 °C. The second subsample was stored at 4 °C and used to determine extracellular enzyme activities and soil physicochemical properties.

### 2.4 Basic soil physicochemical properties and extracellular enzyme activity

Soil ammonium (${\text{NH}}_{4}^{+}$-N) and nitrate (${\text{NO}}_{3}^{-}$-N) were first extracted with 2 M KCl, and their concentrations were then determined colorimetrically using a SEAL Analytical AA3 continuous flow analyzer. Total nitrogen (TN) was measured using the micro-Kjeldahl method, which involved acid digestion of the soil sample followed by distillation and titration. Available phosphorus (AP) was determined using the Olsen method (sodium bicarbonate extraction) followed by molybdenum blue colorimetry, while available potassium (AK) was measured by flame photometry after extraction with ammonium acetate (Liu et al., 2024). Soil pH was measured potentiometrically in a 1:2.5 (w/v) soil-to-water suspension. Soil organic carbon (SOC) was determined by the wet oxidation method using potassium dichromate and sulfuric acid. Soil water content (SWC) was measured gravimetrically by oven-drying fresh soil at 105 °C to a constant weigh (Zhang et al., 2018; Liu et al., 2023).

Determination of soil extracellular enzyme activities related to the C-cycle (β-D-xylosidase, BX; β-D-cellubiosidase, CBH), the N-cycle (N-acetyl-β-glucosaminidase, NAG; leucine aminopeptidase, LAP), and the P-cycle (alkaline phosphatase, ALP) using spectrophotometry. Their activities were quantified by measuring the rate of release of fluorescent or colored products from artificially-labeled substrates (Yang L. et al., 2024).

### 2.5 Wheat quality

During the wheat ripening stage, 50 representative wheat ears were selected from each plot. After threshing and drying, they were ground using a Retsch MM400 ball mill (RETSCH GmbH, Haan, Germany). The crude protein content was determined using the micro-Kjeldahl method, which involves digesting the flour sample, followed by distillation and titration of nitrogen. A nitrogen-to-protein conversion factor of 5.7 was used. Processing quality was determined using a DA7250 near-infrared grain analyzer (Perten Instruments, Stockholm, Sweden), with measurements including wet gluten content and sedimentation value.

### 2.6 Sustainable yield index

The sustainable yield index (SYI) was used to represent yield stability (Wang Z. et al., 2025). Higher SYI values indicate greater yield stability within an agricultural system. The SYI is calculated as follows:


$$\begin{array}{l} SYI=\left(\mu -\sigma \right)/\mu_{\text{max}} \end{array}$$ (1)

where σ, μ, and μ_*max*_ are the standard deviation, and the average value and maximum value of multi-year yields for a specified treatment, respectively.

### 2.7 DNA extraction and sequencing

Total genomic DNA was extracted from soil samples using the E.Z.N.A.^®^ Soil DNA Kit (Omega Bio-tek, USA) following the manufacturer's instructions. DNA quality and concentration were verified by 1.0% agarose gel electrophoresis and NanoDrop 2000 spectrophotometry (Thermo Scientific, USA). For amplicon sequencing, the bacterial 16S rRNA V3-V4 region and fungal ITS1 region were amplified using primers 338F/806R (Caporaso et al., 2012) and ITS1F/ITS2R (Adams et al., 2013), respectively. PCR was performed with the following thermal profile: an initial denaturation at 95 °C for 3 min; 27 cycles of 95 °C for 30 s, 55 °C for 30 s, and 72 °C for 45 s; and a final extension at 72 °C for 10 min. Following amplification, amplicons were gel-purified using a PCR Clean-Up Kit (YuHua, China) and quantified on a Qubit 4.0 fluorometer (Thermo Fisher Scientific, USA). Equimolar amounts of the purified amplicons were pooled to create the final library, which was then subjected to paired-end sequencing on an Illumina Nextseq2000 platform by Majorbio Bio-Pharm Technology Co., Ltd. (Shanghai, China).

After demultiplexing, the resulting sequences were quality filtered with fastp, yielding an average of 14,295 high-quality reads per sample for 16S rRNA and 34,468 reads per sample for ITS1. These sequences were then merged with FLASH. Then, the high-quality sequences were denoied using the DADA2 plugin in the Qiime2 pipeline with the recommended parameters, which provides single-nucleotide resolution based on error profiles within each sample. DADA2-denoised sequences are often referred to as amplicon sequence variants (ASVs). Subsequently, ASVs corresponding to spike-in sequences were filtered out, and reads were quantified. For each sample, a standard curve was constructed by plotting read counts against spike-in DNA copy numbers, which was then used to determine the quantitative abundance of every ASV. ASVs were taxonomically classified using the Naive Bayes consensus classifier within Qiime2 against the SILVA 16S rRNA reference database. Assignment of fungal taxonomy was performed using the UNITE database.

### 2.8 Statistical analyses

Analysis of Variance, complemented by Duncan's *post-hoc* test, was used to assess the variations in yield, quality, soil physicochemical properties, enzyme activities, and alpha diversity indices. The partial least squares path model (PLS-PM) based on the “plspm” package in R was employed to evaluate relationships among soil properties, microbial communities, soil enzyme activity, and crop performance. The visualization and analysis of the microbial co-occurrence network were generated using Gephi.

## 3 Results

### 3.1 Yield and quality

**Figure 2:**
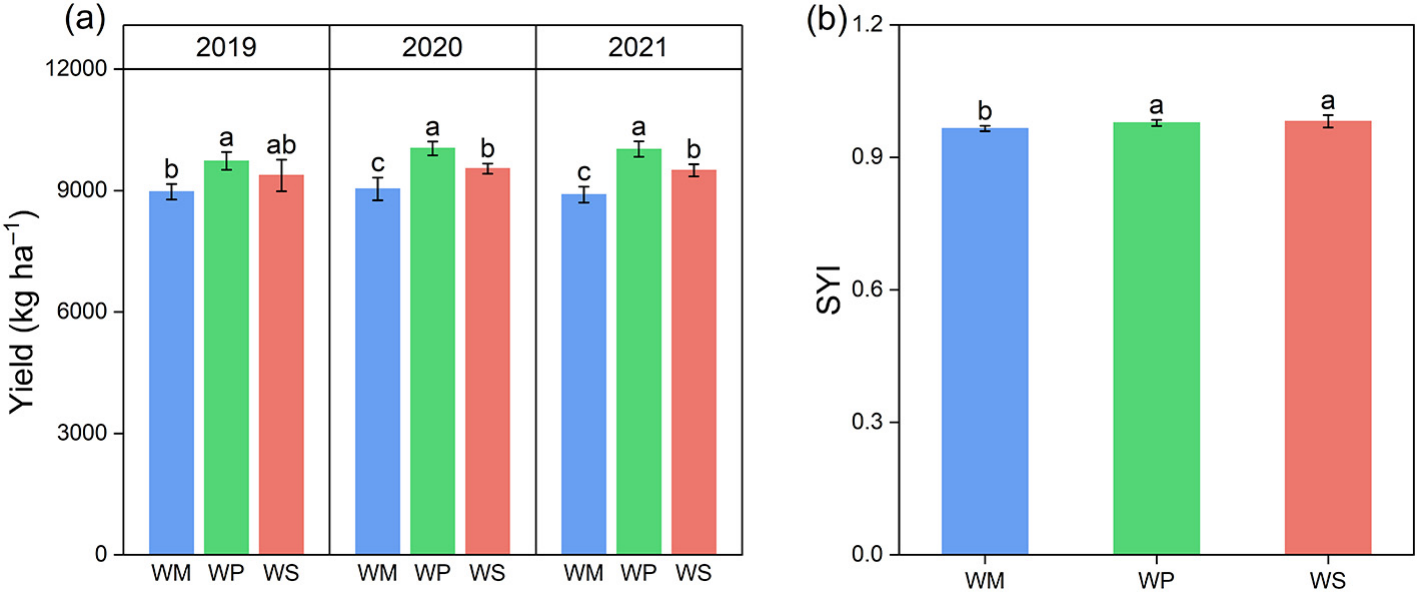
Wheat yield from 2019 to 2021 **(a)** and the yield sustainability index (SYI) **(b)** under different rotation systems. Different lowercase letters above the bars indicate significant differences among rotation treatments within the same season at the *p* < 0.05 level. The data are presented as mean ± SD (*n* = 3). WM, winter wheat-summer maize; WP, winter wheat-summer peanut; WS, winter wheat-summer soybean.

The effect of three crop rotation patterns on wheat yield from 2019 to 2021 is presented in Figure 2a. WP significantly increased wheat yields in all 3 years, averaging 10.7% above WM. In 2019, wheat yield showed no significant difference between the WS and WM. In 2020 and 2021, WS significantly increased wheat yield by 6.1% compared to WM, averaged over 2 years. To quantitatively assess the impact of different cropping systems on yield sustainability, we calculated the yield sustainability index (SYI). Results indicate that crop rotation patterns significantly influenced SYI (*p* < 0.05). Specifically, the WP and WS treatments exhibited significantly higher SYI values than WM, while no significant difference was observed between WP and WS treatments (Figure 2b).

**Figure 3:**
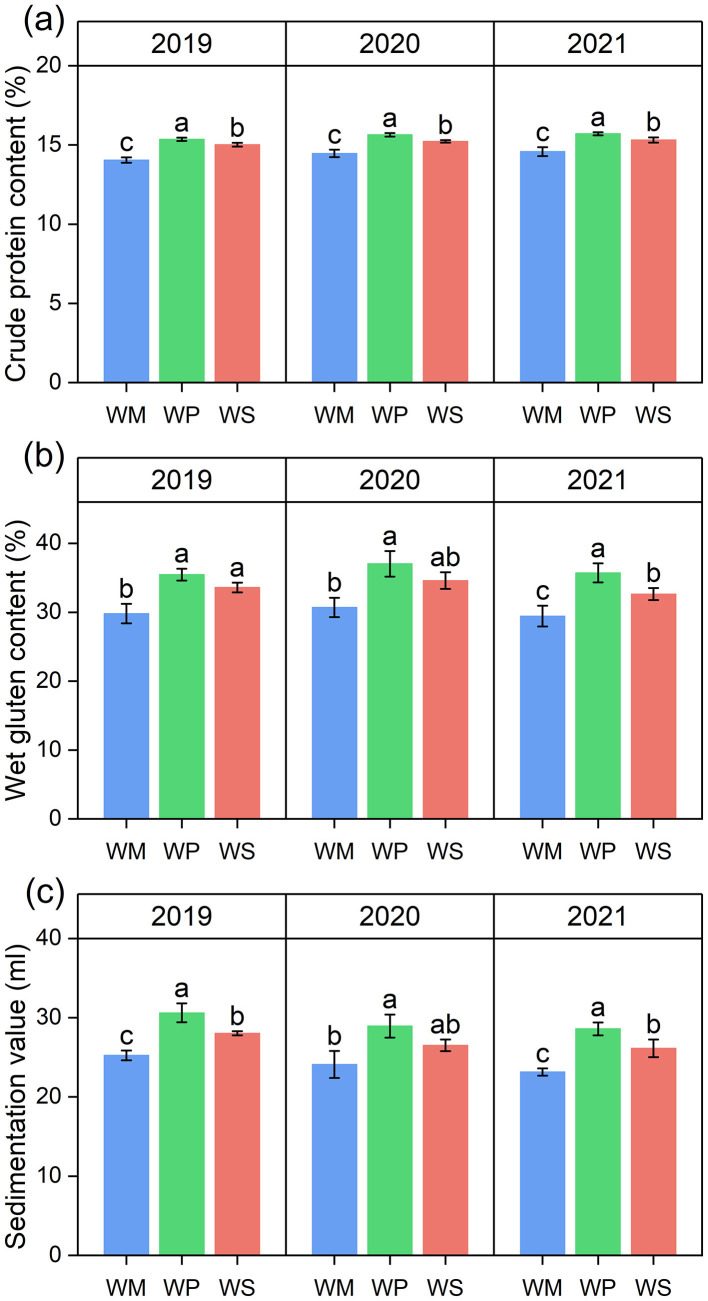
Effects of different rotation systems on crude protein content **(a)**, wet gluten content **(b)**, and sedimentation value **(c)** of wheat grains. Different lowercase letters above the bars indicate significant differences among rotation treatments within the same season at the *p* < 0.05 level. The data are presented as mean ± SD (*n* = 3). WM, winter wheat-summer maize; WP, winter wheat-summer peanut; WS, winter wheat-summer soybean.

Over the three-year study period, the legume-based rotations, particularly WP, consistently and significantly enhanced wheat quality parameters compared to WM (Figure 3). WP consistently delivered the most substantial benefits. On a three-year average, WP increased crude protein by 9.2%, wet gluten by 27.4%, and the sedimentation value by 21.6% relative to WM (Figures 3a–c). WS also improved quality, though to a lesser and more variable extent. It increased average crude protein content by 6.8% compared to WM. However, its effect on other parameters was inconsistent. For example, it showed no significant difference in wet gluten content in 2020, while its average sedimentation value was significantly higher than WM's in all years except 2020.

### 3.2 Soil physicochemical properties

**Figure 4:**
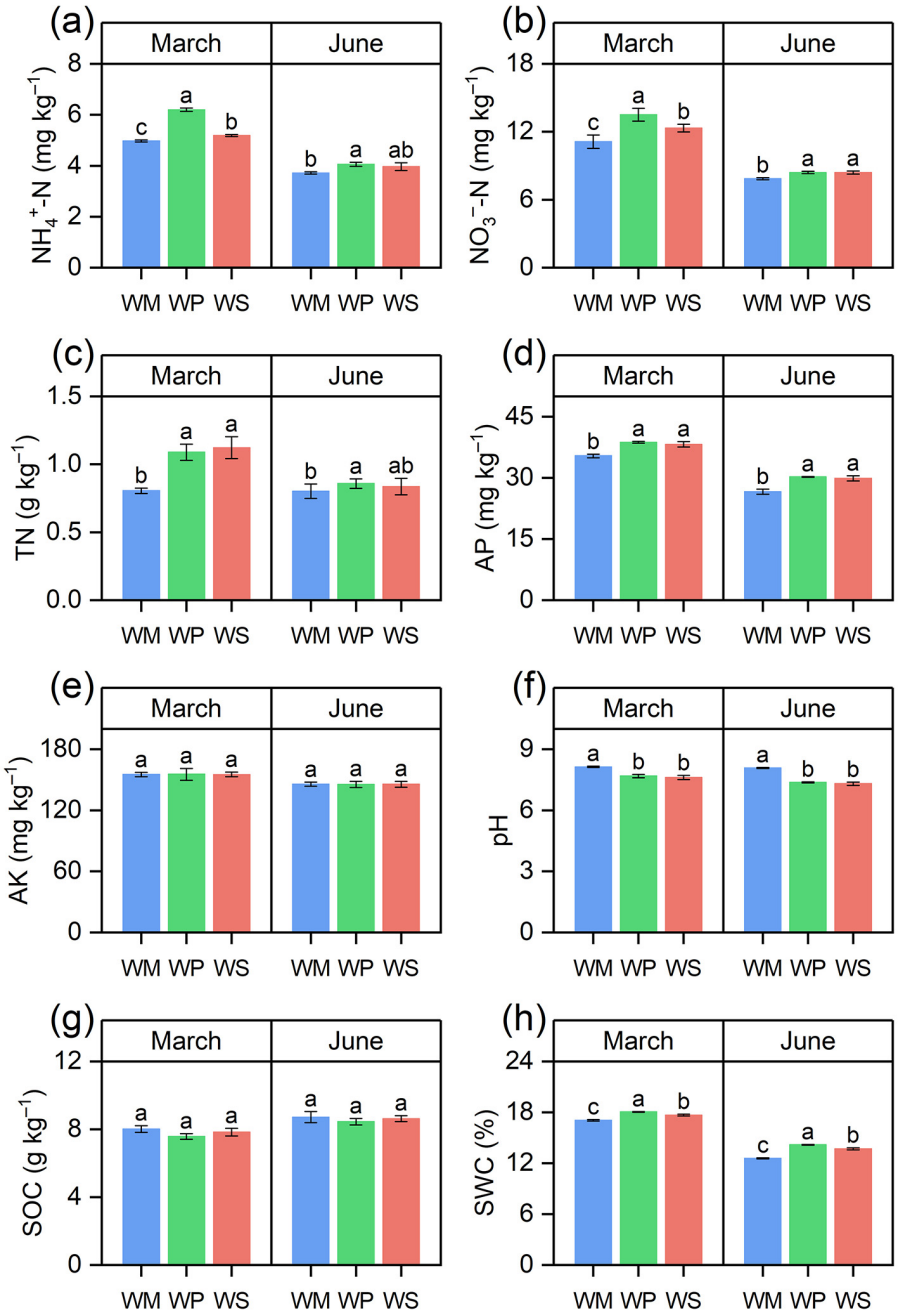
Effects of different rotation systems on soil physicochemical properties. The components in the figure represent: **(a)** ammonium nitrogen (${\text{NH}}_{4}^{+}$-N), **(b)** nitrate nitrogen (${\text{NO}}_{3}^{+}$-N), **(c)** total nitrogen (TN), **(d)** available phosphorus, (AP), **(e)** available potassium (AK), **(f)** pH, **(g)** soil organic carbon (SOC), and **(h)** soil water content (SWC). Different lowercase letters above the bars indicate significant differences among rotation treatments within the same season at the *p* < 0.05 level. The data are presented as mean ± SD (*n* = 3). WM, winter wheat-summer maize; WP, winter wheat-summer peanut; WS, winter wheat-summer soybean.

The introduction of legume crops significantly altered multiple soil physicochemical properties compared to WM, with consistent effects observed across both the March and June sampling seasons (Figure 4). Soil nitrogen dynamics were the most responsive. Compared to WM, WP significantly increased ${\text{NH}}_{4}^{+}$-N content (by 24.7% in March and 9.2% in June) and ${\text{NO}}_{3}^{-}$-N content (by 21.3% and 19.5%, respectively). WS also elevated ${\text{NO}}_{3}^{-}$-N levels by 10.8% in March. Both legume rotations led to higher TN content, with WP showing a 24.0% (March) and 20.6% (June) increase over WM. Furthermore, WP increased AP by 11.3-14.9% across both seasons, whereas AK and SOC were not significantly affected by any rotation pattern. Notably, both WP and WS consistently resulted in a lower soil pH than WM. Regarding soil moisture, WP maintained a significantly higher SWC, with increases of 5.8% (March) and 12.7% (June) over WM. WS also demonstrated superior water-holding capacity.

### 3.3 Soil enzyme activities

The soil extracellular enzyme activities exhibited distinct patterns influenced by both crop rotation and season, with legume-based systems showing a significant shift in nutrient-acquisition strategies compared to WM (Table 1). In contrast to N- and P-cycle enzymes, C-cycle enzyme activities were generally higher under the WM rotation. Legume rotations, however, significantly stimulated N- and P-cycle enzymes. In March, compared to WM, LAP activity increased by 11.0% in WP and 6.2% in WS, while NAG activity rose by 14.1% and 8.4%, respectively. This enhancement in N-cycle enzyme activities was also observed in June. For P-cycle, ALP activity was similarly higher in both legume systems than in WM during March.

**Table 1:** The soil extracellular enzyme activities under different rotation systems.

**Soil enzyme**	**March**			**June**		
	**WM**	**WP**	**WS**	**WM**	**WP**	**WS**
BX (U g^−1^)	673.30a	649.26b	653.87ab	365.91a	352.64a	354.03a
CBH (U g^−1^)	92.78a	85.89b	86.63b	56.77a	53.51b	53.40b
LAP (U g^−1^)	590.69c	655.83a	627.14b	493.30c	570.38a	541.03b
NAG (U g^−1^)	162.41c	185.24a	176.04b	154.27c	171.49a	161.96b
ALP (U g^−1^)	9.61b	10.91a	10.81a	11.22b	12.52a	12.26a

WM, winter wheat-summer maize; WP, winter wheat-summer peanut; WS, winter wheat-summer soybean; BX, β-xylosidase; CBH, 1, 4-β-cellobiohydrolase; LAP, L-Leucine ami-nopeptidase; NAG, N-acetylglucosaminidase; ALP, alkaline phosphatase. Different lowercase letters indicate significant differences at *p* < 0.05.

### 3.4 Soil microbial community composition

**Figure 5:**
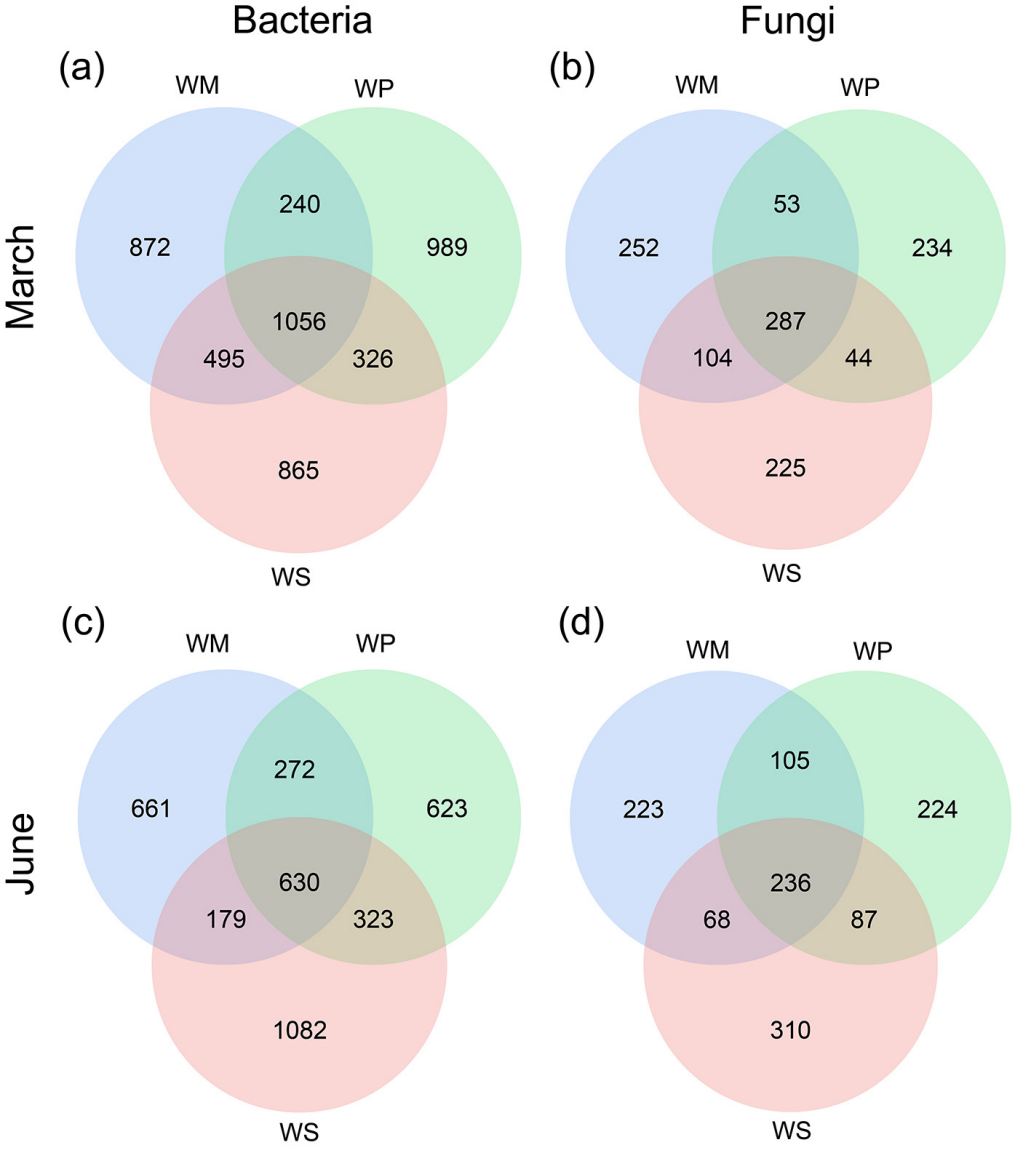
The Venn diagram showing the unique and shared ASVs among three rotation patterns. **(a)** Bacterial communities in March. **(b)** Fungal communities in March. **(c)** Bacterial communities in June. **(d)** Fungal communities in June. WM, winter wheat-summer maize; WP, winter wheat-summer peanut; WS, winter wheat-summer soybean.

Venn diagrams (Figure 5) were used to visualize the shared and unique ASVs in the bacterial and fungal communities across the three rotation patterns at two key growth stages (March and June). For the bacterial communities (Figures 5a, c), the number of shared ASVs common to all three rotation patterns was larger in March (1056 ASVs) than in June (630 ASVs), indicating a more conserved core microbiome at the earlier growth stage. In March, the highest number of unique bacterial ASVs was observed in WP (989). The WM and WS showed lower richness, with 872 and 865 ASVs, respectively. For the fungal communities (Figures 5b, d), a similar pattern was observed. The number of shared ASVs common to all three rotation patterns was larger in March (287 ASVs) than in June (236 ASVs). However, the distribution of unique ASVs showed notable system-specificity. WP consistently had fewer unique fungal ASVs in both March (234) and June (224).

**Figure 6:**
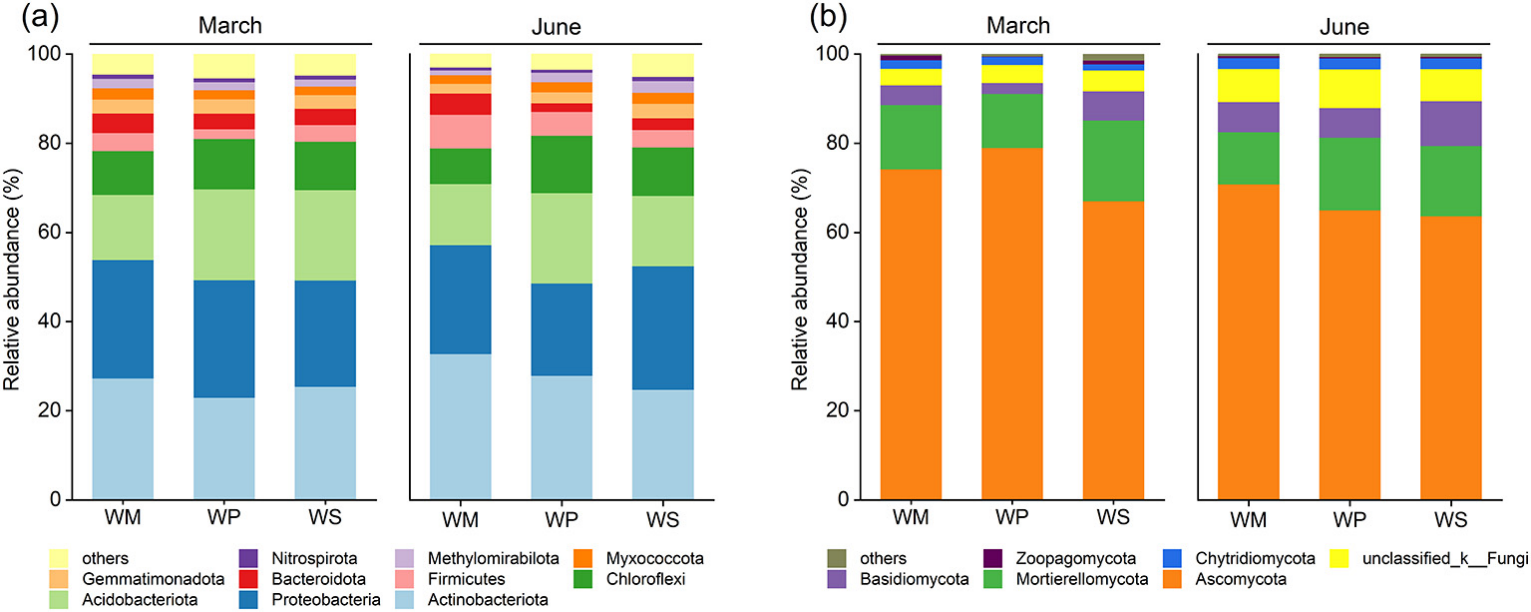
Relative abundance of dominant bacterial **(a)** and fungal **(b)** phyla. WM, winter wheat-summer maize; WP, winter wheat-summer peanut; WS, winter wheat-summer soybean.

Shifts in the relative abundance of dominant bacterial and fungal phyla in soils sampled in March and June were assessed to determine the impact of crop rotation patterns during the wheat season (Figure 6). Proteobacteria, Actinobacteria, Acidobacteria, Chloroflexi, and Gemmatimonadota were the dominant phyla at the bacterial level across all samples. Compared to WM, the legume-based rotations consistently increased the relative abundances of Acidobacteria and Chloroflexi across both sampling seasons. Specifically, the abundance of Acidobacteria under WP was 20.34% in March and 20.21% in June, which were higher than the corresponding values in WM (14.58% and 13.68%). Similarly, Chloroflexi was more abundant in WP (11.28% in March, 12.89% in June) than in WM (9.80% in March, 8.02% in June). The relative abundance of Actinobacteria was reduced in the legume systems, particularly in June, when it was lower in WP (27.93%) and WS (24.77%) than in WM (32.80%). The response of Proteobacteria differed between the two legume crops: in June, its abundance decreased in WP (20.72%) relative to WM (24.43%), but increased in WS (27.71%). At the fungal phylum level, the Ascomycota phylum dominated all samples and constituted the core component of the fungal community. The Mortierellomycota and Basidiomycota phyla followed it. The abundance of Ascomycota exhibited significant phasic and treatment-specific variations. The highest and lowest abundances of Ascomycota in March were detected in the WP and WS treatments, respectively. By June, the WM showed the highest abundance of Ascomycota, whereas both legume rotation treatments (WP and WS) exhibited a decline in abundance. Unlike the Ascomycota, the relative abundance of the Basidiomycota consistently elevated across all treatments in June.

### 3.5 Soil microbial community diversity

**Figure 7:**
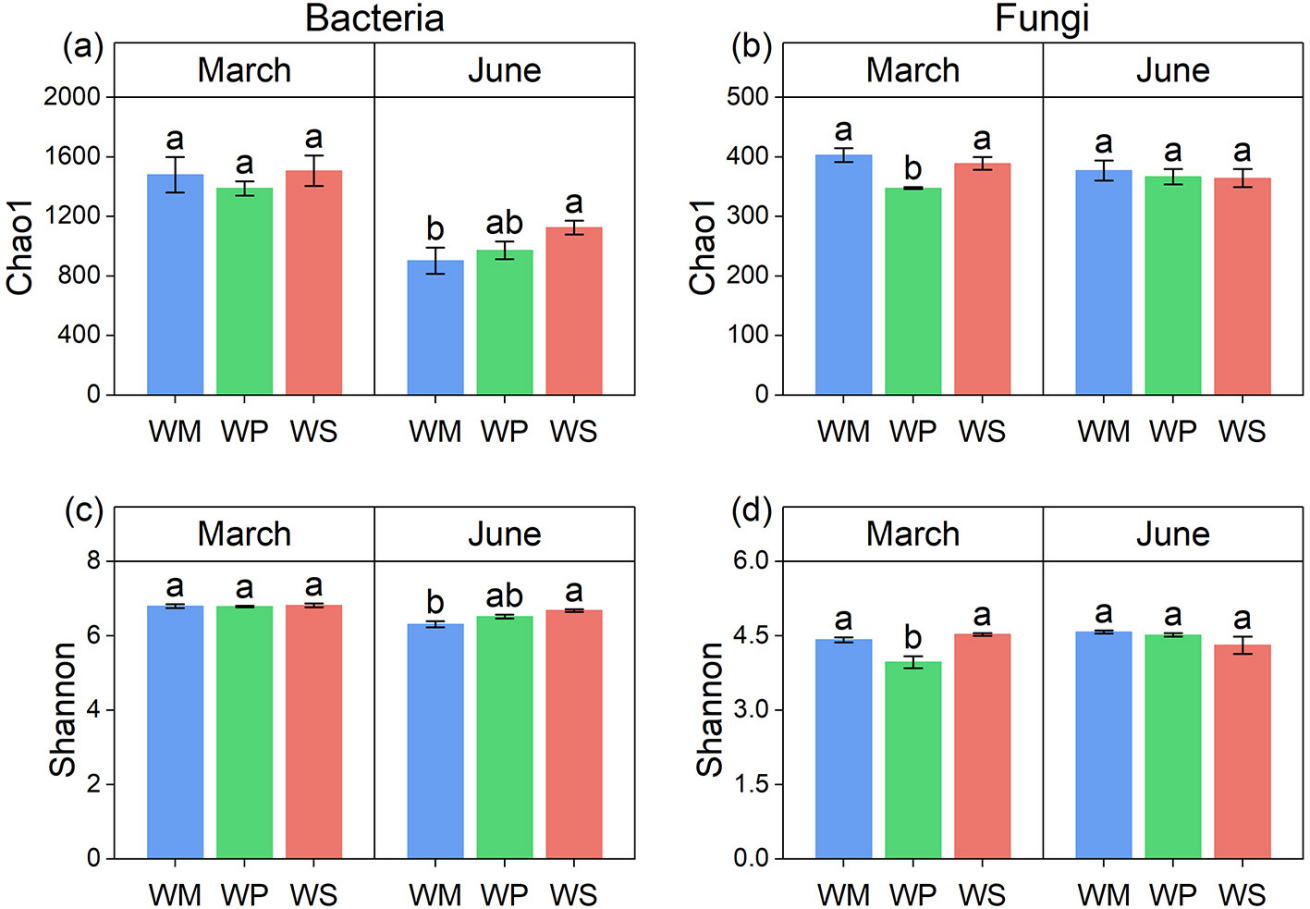
Alpha diversity indices of soil microbial communities under different crop rotation patterns. **(a)** Chao1 richness index for bacterial communities. **(b)** Chao1 richness index for fungal communities. **(c)** Shannon diversity index for bacterial communities. **(d)** Shannon diversity index for fungal communities. Different lowercase letters above the bars indicate significant differences among rotation treatments within the same season at the *p* < 0.05 level. WM, winter wheat-summer maize; WP, winter wheat-summer peanut; WS, winter wheat-summer soybean.

**Figure 8:**
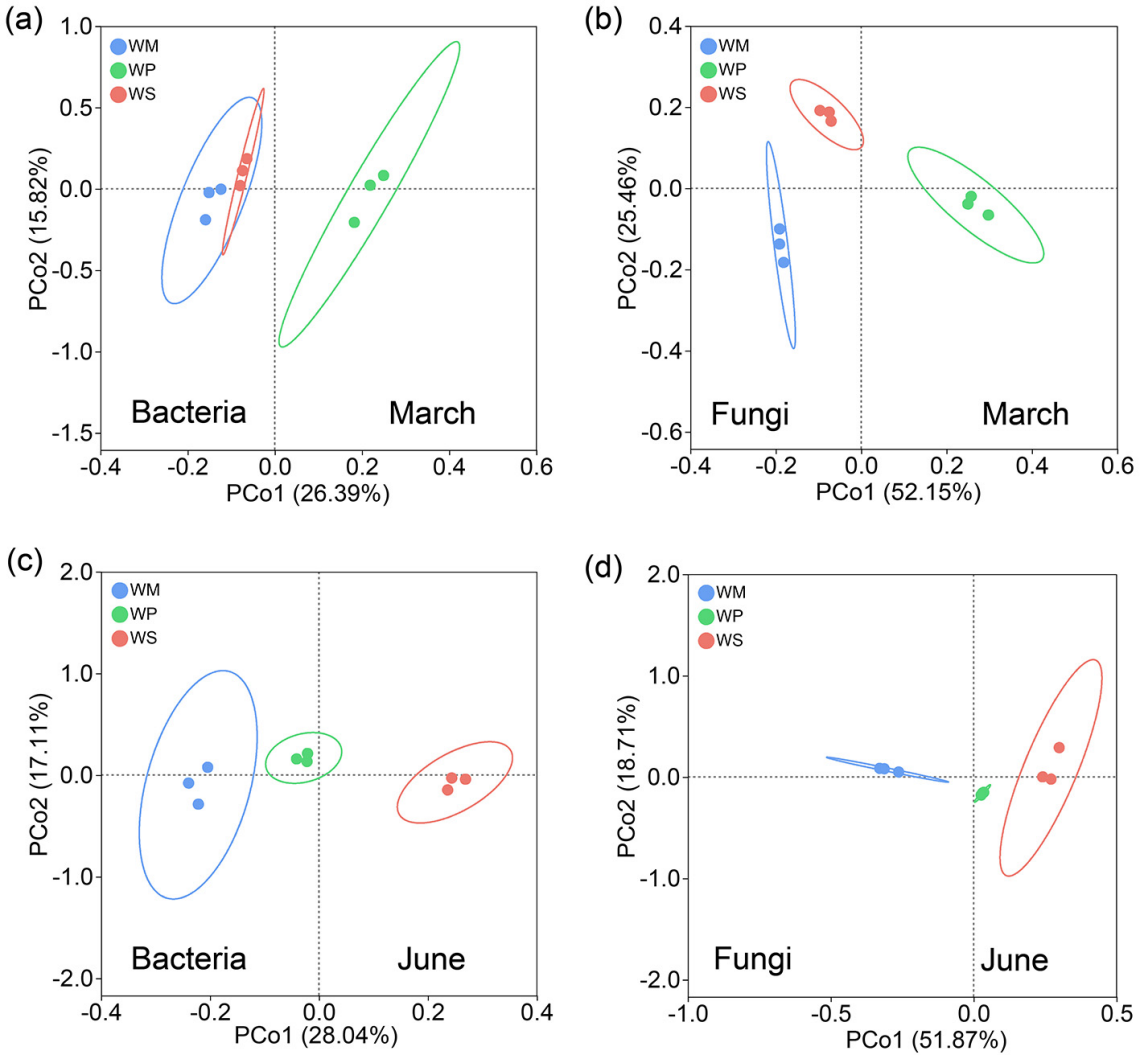
Principal Coordinate Analysis (PCoA) of microbial communities under different crop rotation patterns, showing bacterial **(a)** and fungal **(b)** communities in March, and bacterial **(c)** and fungal **(d)** communities in June. WM, winter wheat-summer maize; WP, winter wheat-summer peanut; WS, winter wheat-summer soybean.

Analysis of Chao1 richness and Shannon diversity indices revealed that the alpha diversity of soil microbial communities was differentially affected by both rotation pattern and sampling season (Figure 7). For bacterial communities, the cropping system had a season-dependent effect. At the March sampling, no significant differences in either bacterial richness (Chao1, Figure 7a) or diversity (Shannon, Figure 7b) were observed among the three rotation patterns. However, by June, a clear hierarchy emerged, with both indices showing a consistent pattern of WS > WP > WM. The fungal communities exhibited a distinctly different response pattern. In March, significant differences were observed, with WP showing significantly lower values in both fungal richness (Chao1, Figure 7c) and diversity (Shannon, Figure 7d) compared to WM and WS. These differences, however, diminished by June, with all three cropping systems maintaining statistically similar levels of fungal alpha diversity at the later stage. The three cropping systems significantly shaped both bacterial and fungal community structures, leading to clear separations on the PCoA plot across both sampling seasons (Figure 8).

### 3.6 Soil microbial co-occurrence network analysis

**Figure 9:**
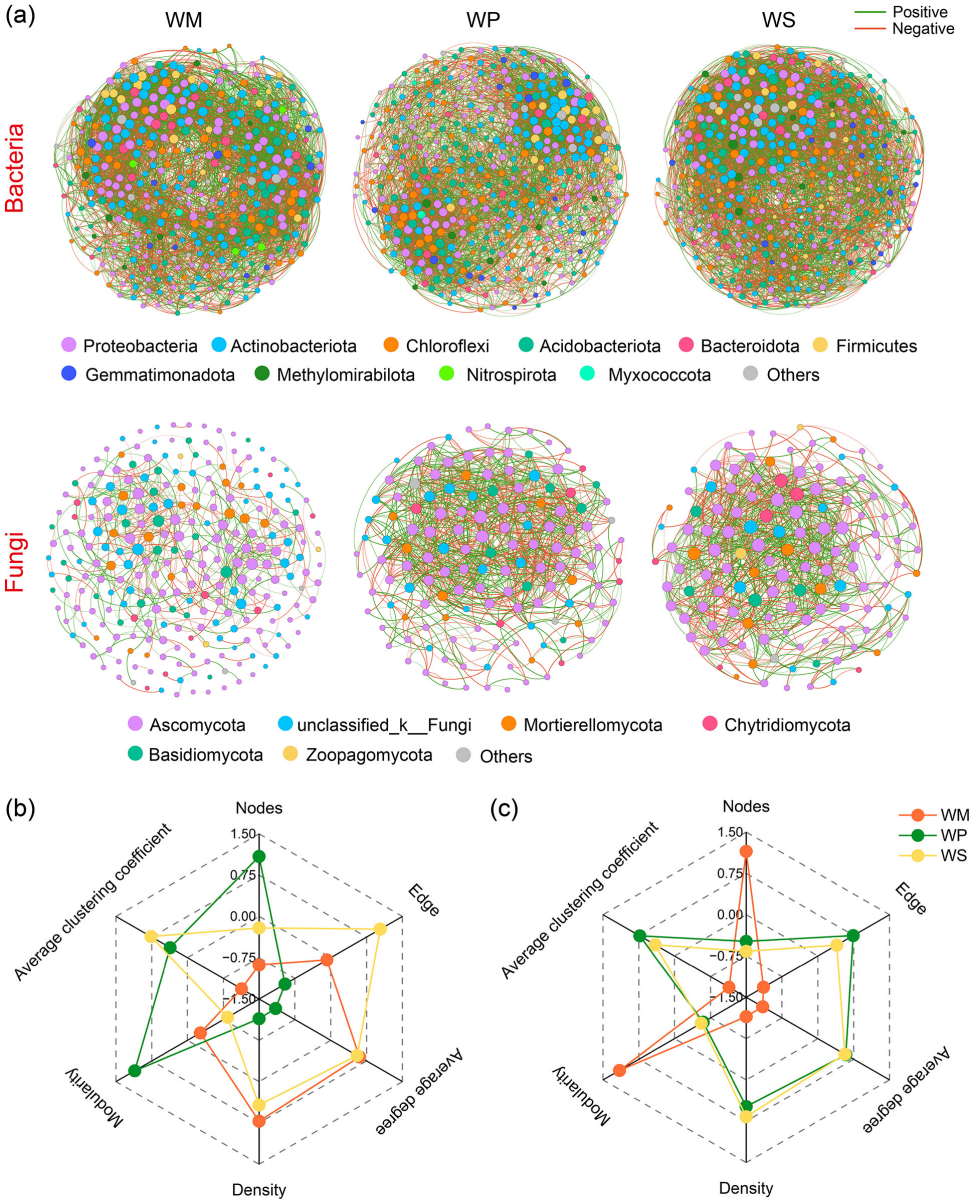
Bacterial and fungal co-occurrence networks under different rotation systems **(a)**. Topological properties of empirical networks of bacteria **(b)** and fungi **(c)** under different rotation systems. WM, winter wheat-summer maize; WP, winter wheat-summer peanut; WS, winter wheat-summer soybean.

Figure 9 demonstrates that crop rotation systems exerted a strong influence on the structure of both bacterial and fungal co-occurrence networks. In bacterial networks, legume rotations (WP and WS) exhibit larger but sparser network structures compared to WM. Specifically, both WP and WS have more nodes than WM, yet their average degree and network density are lower. Among these, the WP network contained the highest number of nodes but the lowest number of edges, and exhibited the highest modularity, indicating the most stable community structure. Additionally, both legume rotation networks possessed higher average clustering coefficients than WM, suggesting their nodes tended to form tighter local clusters. The fungal networks responded more dramatically to the legume-based rotations. Both WP and WS substantially increased the network complexity compared to WM. Compared to the WM network, the fungal networks under WP and WS were more complex, featuring higher average degrees, a greater number of links, and larger proportions of both positive and negative interactions. Concurrently, these more intricate networks exhibited lower modularity and a shorter average path distance. Notably, the average clustering coefficient was also significantly higher in the legume systems, with WP showing the highest value.

### 3.7 Analysis of correlations

**Figure 10:**
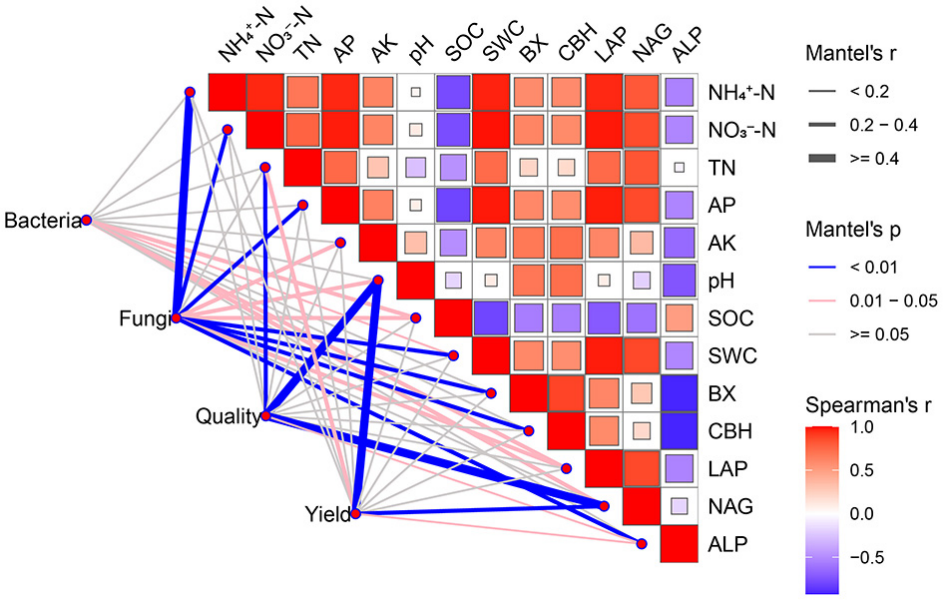
The relationship of soil physicochemical properties and extracellular enzyme activities with bacterial and fungal communities by Mantel tests. Pairwise comparisons of soil physicochemical properties and extracellular enzyme activities were represented by rectangular boxes, with red for positive and blue for negative correlations. Edge width indicates the coefficients of the partial Mantel tests, while edge color represents correlation significance. WM, winter wheat-summer maize; WP, winter wheat-summer peanut; WS, winter wheat-summer soybean.

Mantel test analysis revealed significant correlations between wheat yield and quality and key soil variables, including soil pH, TN, NAG, and ALP. The bacterial community structure exhibited significant correlations with SOC, AP, SWC, LAP, and NAG. The fungal communities were strongly associated with most soil factors (Figure 10).

### 3.8 PLS-PM analysis

**Figure 11:**
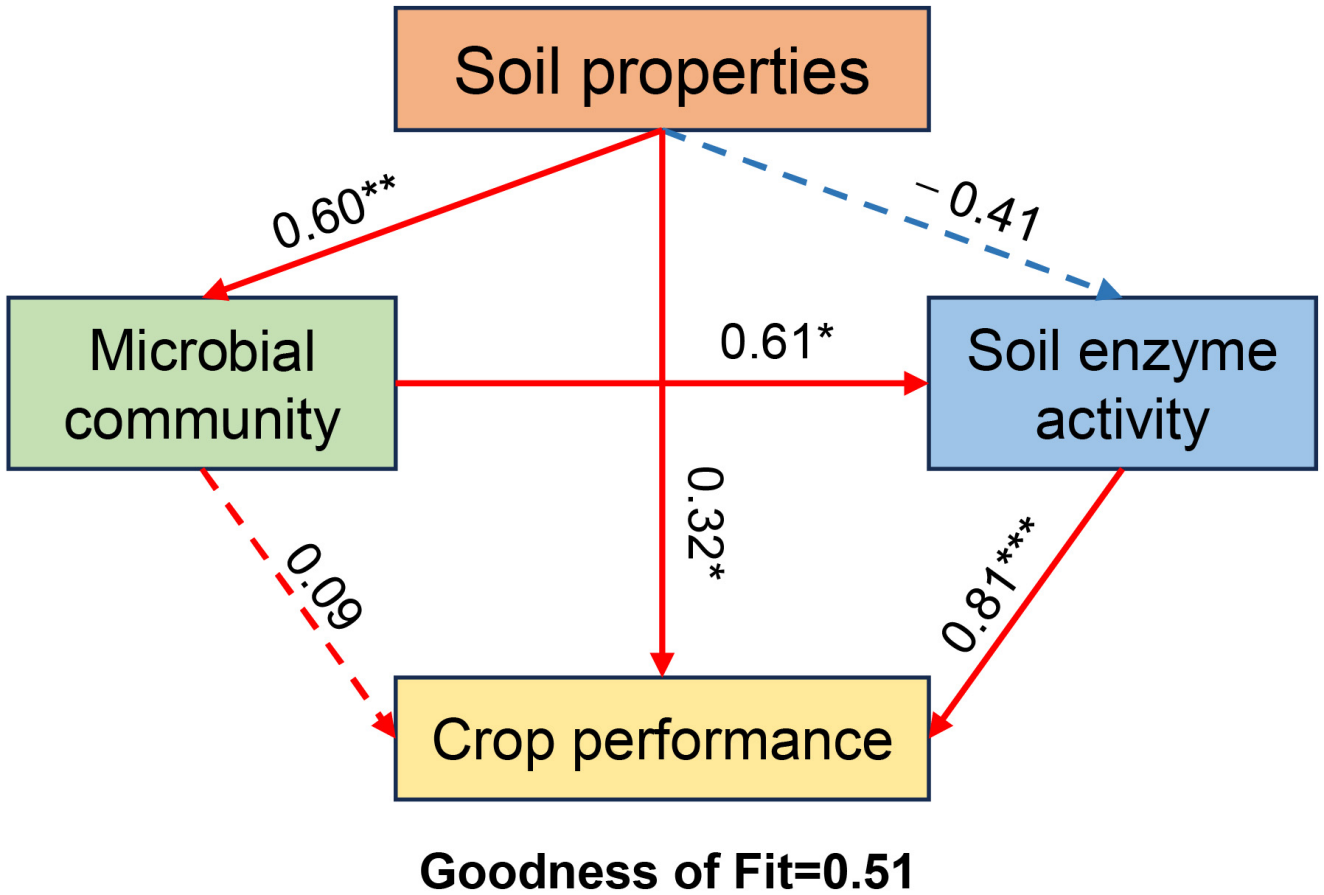
Partial least squares path model (PLS-PM) analysis of the relationships among soil properties, microbial communities, soil enzyme activity, and crop performance. Positive and negative effects were represented by red and blue arrows, respectively. Path coefficients with no significant difference were indicated by dashed lines. Significance is indicated by *p < 0.05, **p < 0.01, and *** *p* < 0.001.

A partial least squares path model (PLS-PM) was constructed to identify key factors driving crop performance (Figure 11). Soil enzyme activity exerted the strongest direct positive effect on crop performance, reaching a highly significant level, making it the most crucial direct determinant of final crop performance. Additionally, soil properties significantly and positively influenced crop performance. As the most fundamental driving factor in the system, soil properties significantly promoted microbial community assembly. Improvements in microbial communities further significantly elevated soil enzyme activity. Overall, soil properties not only directly enhance crop performance but, more importantly, exert a more potent influence through a key indirect pathway: soil properties first affect microbial communities, which in turn alter soil enzyme activity. This demonstrates that microbial communities and soil enzyme activity play crucial mediating roles ithin this system.

## 4 Discussion

### 4.1 Effects of legume-based rotation on soil physicochemical properties and biological properties

Legumes not only satisfy their own nitrogen requirements through biological nitrogen fixation, but also provide a nitrogen source for subsequent crops (Geng et al., 2023). In this study, both legume systems, particularly WP, significantly increased ${\text{NH}}_{4}^{+}$-N and ${\text{NO}}_{3}^{-}$-N during the wheat season (Figure 4). Legumes have been shown to store a portion of nitrogen in their root systems and nodules, which is released into the soil through rhizosphere exudates and the turnover of roots and nodules (Hupe et al., 2019; Geng et al., 2024). This also explains the increased N availability during the wheat season in this experiment. Notably, this enhancement effect is particularly pronounced in WP. This may be because peanut straw has a low C/N ratio, accounting for 40% of the plant's total nitrogen, which is beneficial for residue decomposition and net nitrogen mineralization (Huang et al., 2025). This study found that compared to WM, WP significantly increased SWC and AP levels in the subsequent wheat season. The increase in SWC is primarily attributed to peanuts' lower water consumption compared to maize. The rise in AP might be due to the fact that, as a legume, peanut roots secrete organic acids and phosphatase, effectively activating immobilized insoluble phosphorus and inorganic phosphorus in the rhizosphere soil (Chen et al., 2025). Soil pH is a core variable among numerous factors influencing soil fertility, as it determines nutrient solubility, microbial community structure, and enzyme activity (Jin et al., 2022; Duan et al., 2025). This study found that, compared to WM, both WP and WS significantly decreased soil pH, which may be attributed to legume root systems secreting organic acids (such as citric and malic acids) to release phosphorus that would otherwise be immobilized in the soil (Nuruzzaman et al., 2005). Comparable findings were reported in a study on legume-potato rotation (Wang et al., 2023b). Several studies have reported that legume-based crop rotations can increase SOC sequestration (Yang X. et al., 2024; Liu W. et al., 2025). However, we found no significant difference in SOC content between the legume rotation and the maize-wheat rotation, possibly because SOC changes are slow and the 3 year trial period may be insufficient to capture such gradual shifts (Lasisi and Liu, 2023). Therefore, longer-term investigations should be conducted to validate the efficacy of legume rotation systems for enhancing soil organic carbon.

Soil extracellular enzymes serve as the direct tools through which microorganisms participate in nutrient cycling (Li et al., 2024). Changes in their activity profiles reveal fundamental shifts in microbial nutrient acquisition strategies under different crop rotation systems. This study found that the WM system exhibited higher C-cycle enzyme activity, while the legume rotation system significantly stimulated the activity of N- and P-cycle enzymes (Table 1). WM system introduced straw with a high C/N, causing microorganisms to be N-limited during decomposition and thus prioritizing the synthesis of C-cycle enzymes (Wang et al., 2015). Conversely, the low C/N ratio residues introduced by legume rotation were rich in nitrogen, greatly stimulating microbial activity (Sui et al., 2025). To meet their own rapid growth demands, microbial communities strategically upregulated the activity of nitrogen and phosphorus cycling enzymes to mineralize these key nutrients from soil organic matter (Allison et al., 2007). This process contributes to promote subsequent higher yields (Yan et al., 2024).

### 4.2 Effects of legume-based rotation on soil microbial community composition and network characteristics

The findings of this study suggest that, from March to June, the number of core bacterial and fungal ASVs significantly decreased across all rotation patterns (Figure 5). This finding indicates that as wheat plants grew and root exudates changed, the soil microbial community underwent a succession process from a relatively generalized legacy community to a more selective and specific rhizosphere community. This shift aligns with prior research indicating that crop growth stages are primary determinants of microbial community structure evolution (Chen et al., 2019). Legume rotation altered the relative abundance of key microbial phyla. Both WP and WS consistently increased the abundance of slow-growing, oligotrophic bacteria. Acidobacteria specialize in breaking down complex organic matter (Eichorst et al., 2011). Chloroflexota promotes the dissolution of citrate phosphate, indirectly supporting nitrogen absorption (Cao et al., 2017). By fixing nitrogen and leaving decomposable residues, legume crops create a favorable environment for these bacteria (Liu Y. et al., 2025).

Ascomycota constitutes the predominant phylum in the fungal community (Shen et al., 2020). The phenomenon we observed in March, in which peanut (WP) promoted and soybean (WS) inhibited this phylum, may be attributed to chemical differences in root exudates or early decomposition products between peanut and soybean (Zhalnina et al., 2018). These differences could have specifically recruited or suppressed distinct functional subgroups within Ascomycota (Sasse et al., 2018). By June, however, both legume rotations suppressed the phylum Ascomycota, while the WM maintained its high abundance. We speculate that this is related to the high-nitrogen environment in legume rotations, which inhibits certain saprophytic Ascomycota required to decompose organic matter with a high C/N ratio (Purahong et al., 2016). A significant finding was the consistent increase in the relative abundance of the Basidiomycota within all treatments in June (Figure 6). Basidiomycota are considered the most efficient lignin decomposers in ecosystems (Voríšková and Baldrian, 2013). The increase indicates that at the late stage of the wheat growing season, with the depletion of labile carbon sources, the metabolic priority of the soil microbial community shifts toward the decomposition of more recalcitrant plant matter, such as senescent root systems and surface stubble.

By constructing a co-occurrence network, we gained further insight into how different crop rotation schemes shape the microbial community structure (Figure 9). Legume rotations had distinctly different effects on bacterial and fungal networks. For the bacterial network, even though the WP contained the maximum number of nodes, it had the fewest edges and the lowest average degree, suggesting a more modular network. This network structure may imply that while the bacterial community in WP is species-rich, its interspecific interactions are relatively fragile or straightforward (Sun et al., 2025). Elevated biodiversity does not inherently lead to greater network intricacy and robustness (Zhang et al., 2024). In resource-rich environments, a microbial network with reduced complexity may still exhibit high functional redundancy (Yu et al., 2024). In the fungal network, legume rotations (both WP and WS) greatly increased the network complexity. Concurrently, decreases in network modularity and in the average path distance suggest that the fungal community became more interconnected and efficient (Tylianakis et al., 2010). Notably, the highest average clustering coefficient under WP indicates the formation of more tightly knit clusters with overlapping niches within its fungal community (Felipe-Lucia et al., 2020). Such a complex and highly connected fungal network is generally considered to possess stronger resistance and resilience, as well as higher nutrient cycling efficiency (Morriën et al., 2017). These beneficial properties could be among the key mechanisms by which legume rotations enhance soil health and functionality.

### 4.3 Effects of legume-based crop rotation on wheat yield and quality

The goal of optimizing cropping systems is to improve crop yield and grain quality. Our study unequivocally showed that legume-based rotations, particularly the WP system, significantly improved wheat performance compared to the conventional WM system. The WP rotation achieved a consistent yield increase and substantially improved nutritional quality, with crude protein and wet gluten content increasing (Figures 2, 3). These improvements result from the synergistic effects of the optimized soil physicochemical environment and the reshaped microbial functions discussed in the previous sections (Guo et al., 2024). First, the enhancement in yield and quality is directly underpinned by improved nutrient availability. As the chief constraint for wheat yield, nitrogen is also the essential building block for grain protein and gluten accumulation (Maaz et al., 2021; Wang et al., 2023a). Legume crop rotation leaves behind a nitrogen legacy, characterized by elevated levels of TN, ${\text{NH}}_{4}^{+}$-N, and ${\text{NO}}_{3}^{-}$-N (Figure 4). This abundant N supply provided the material basis for the observed surge in protein and gluten content (Zörb et al., 2018). Simultaneously, legume rotation increases soil phosphorus availability. Since phosphorus is vital for energy transfer and grain filling, this increased availability likely contributed to the higher sedimentation values and overall yield (Ahmadi et al., 2024). The Mantel test analysis further corroborated this mechanism, identifying TN, soil pH, and AP as key environmental drivers significantly correlated with wheat yield and quality (Figure 10). Second, the strategic shift in soil enzymatic activity catalyzed the conversion of soil nutrients into plant-available forms. A strong positive correlation was observed between wheat quality and the activities of N- and P-acquiring enzymes, as determined by a Mantel test (Figure 10). This finding suggests that the legume-induced upregulation of these specific enzymes effectively accelerated the mineralization of organic N and P, matching nutrient supply with the requirements of wheat at key developmental phases (Zhang L. et al., 2025). The superior performance of the WP over the WS in terms of yield and quality can be partly attributed to greater increases in these enzymatic activities and nutrient levels in peanut stubble (Zhang et al., 2022). The stability of the soil microbial ecosystem likely played an indirect but vital role in sustaining high productivity (Long et al., 2025). As highlighted in Figure 9, WP fostered a fungal network with higher complexity and connectivity. A robust fungal network is often associated with improved nutrient transport efficiency and enhanced crop resilience to environmental stresses (Zhang Z. et al., 2025). Combined with the higher SWC observed in legume rotations, this created a more buffered and supportive soil environment, ensuring that the wheat crop could fully realize its yield and quality potential even under varying seasonal conditions. Our study revealed the impacts of different rotation systems on sustainability of wheat yield. The results indicated that legume-wheat rotation system exhibited greater yield sustainability index (YSI). One reason is that higher crop yields can lead to more consistent production, thereby reducing the impact of meteorological variations from 1 year to the next (Lin et al., 2026). Another possible reason is that improved soil enzyme activity and nutrient availability under legume rotation treatments enhanced crop stress tolerance and reduced yield fluctuations (Macholdt et al., 2019).

### 4.4 Relationships among soil properties, microbial communities, soil enzyme activity, and crop performance

Utilizing a partial least squares path model (PLS-PM), this study elucidated the key pathways driving crop yield and quality across different rotation systems (Figure 11). Soil enzyme activity as the primary factor directly influencing crop performance. As the catalysts for material cycling and nutrient transformation, soil enzymes govern the efficiency of organic matter decomposition and nutrient mineralization, thereby ensuring a sustained supply of plant-available nutrients (Smutny et al., 2025). Consequently, performance differences among rotation systems could be largely attributed to these variations in soil enzyme activity. Furthermore, the model delineated a critical indirect pathway through which rotation system impacts crop performance. This cascade begins with soil physicochemical properties, which in turn shape the microbial community, subsequently regulating soil enzyme activity, and ultimately affecting crop performance. Different rotation patterns, particularly the inclusion of legumes, altered soil physicochemical properties via processes like root exudation, litter input, and biological nitrogen fixation (Yan et al., 2024; Zhang L. et al., 2025). These properties then selectively structured the soil microbial community. As the main producers of soil enzymes, the microbial community's specific composition and function ultimately determined the overall level of enzyme activity (Hansen et al., 2026). Notably, while soil physicochemical properties exerted a significant direct effect on crop performance, their indirect effects that were mediated through the microbial community and soil enzyme activity proved to be of greater magnitude. This indicates that the microbial community and its enzymatic functions are the crucial mediating variables in this system, serving as the primary link between the soil environment and crop output (Hansen et al., 2026).

## Conclusions

This study demonstrates that incorporating legume, particularly peanut, into wheat rotation system significantly enhances wheat yield and quality compared to wheat-maize rotation. The wheat-peanut rotation was the most effective, achieving a 10.7% yield increase and a 27.4% boost in wet gluten content. These benefits stem from a holistic improvement in soil health, as legumes increase nitrogen availability and mobilize phosphorus by enhancing nutrient-cycling enzyme activities and fostering a more complex, resilient fungal network. Consequently, the wheat-peanut rotation offers a sustainable strategy to optimize both crop productivity and soil ecosystem function.

## Data Availability

The original contributions presented in the study are publicly available. This data can be found at: https://www.ncbi.nlm.nih.gov/, accession number PRJNA1402120.
